# Supplementary material for: First Detection and Genetic Characterization of Swine Orthopneumovirus from Domestic Pig Farms in the Republic of Korea
Source: Viruses. 2023 Nov 30;15(12):2371. doi: 10.3390/v15122371 (PMC10747143; doi:10.3390/v15122371)
Supplement: Supplementary file 1 [file viruses-15-02371-s001.zip › Supplementary data - final.pdf]

Supplement Data

Table S1. Details of the oral fluid samples.

Province	Farm	Age
Gangwon-do	GW1	1 weeks, 3 weeks
Gangwon-do	GW2	4 weeks
Gangwon-do	GW3	2 weeks
Gyeonggi-do	GG1	4 weeks
Gyeonggi-do	GG2	4 weeks
Gyeonggi-do	GG3	6 weeks
Gyeonggi-do	GG4	1 weeks
Gyeonggi-do	GG5	3 weeks
Chungcheongbuk-do	CB1	1 weeks
Chungcheongnam-do	CN1	5 weeks, 8 weeks, 9 weeks
Chungcheongnam-do	CN2	1 weeks
Chungcheongnam-do	CN3	2 weeks
Chungcheongnam-do	CN4	16 weeks
Jeollabuk-do	JB1	4 weeks, 10 weeks, 13 weeks, 16 weeks, 22 weeks, reserve sows
Jeollabuk-do	JB2	4 weeks, 5 weeks, 6 weeks, 7 weeks, 9 weeks
Jeollabuk-do	JB3	4 weeks, 8 weeks
Jeollabuk-do	JB4	4 weeks, 6 weeks, 8 weeks, 10 weeks
Jeollabuk-do	JB5	20 weeks
Jeollabuk-do	JB6	4 weeks, 6 weeks, 8 weeks, 10 weeks, 13 weeks, 16 weeks, 19 weeks, 22 weeks, reserve sows, farrowing sows
Jeollabuk-do	JB7	11 weeks, 12 weeks
Jeollanam-do	JN1	1 weeks, 5 weeks, 10 weeks, 15 weeks, 24 weeks, 30 weeks, 42 weeks, farrowing sows
Jeollanam-do	JN2	4 weeks
Jeollanam-do	JN3	4 weeks
Jeollanam-do	JN4	8 weeks, 9 weeks, 10 weeks, 11 weeks, 12 weeks
Jeollanam-do	JN5	4 weeks, 6 weeks, 7 weeks, 8 weeks, 9 weeks, 10 weeks, reserve sows, farrowing sows
Gyeongsangbuk-do	GB1	2 weeks, 4 weeks, 10 weeks
Gyeongsangbuk-do	GB2	6 weeks
Gyeongsangbuk-do	GB3	12 weeks
Gyeongsangbuk-do	GB4	6 weeks, 12 weeks
Gyeongsangbuk-do	GB5	4 weeks, 6 weeks, 8 weeks, 10 weeks, farrowing sows
Gyeongsangbuk-do	GB6	4 weeks, 6 weeks, 8 weeks, 10 weeks

Gyeongsangbuk-do	GB7	reserve sows, farrowing sows
Gyeongsangbuk-do	GB8	3 weeks, 7 weeks, 9 weeks, 13 weeks
Gyeongsangnam-do	GN1	reserve sows
Gyeongsangnam-do	GN2	8 weeks
Gyeongsangnam-do	GN3	2 weeks
Gyeongsangnam-do	GN4	7 weeks
Gyeongsangnam-do	GN5	11 weeks
Gyeongsangnam-do	GN6	4 weeks
Jeju-do	JJ1	2 weeks, 4 weeks

Table S2. Primer sets for the PCR of complete genome and G gene in this study.

Target	Primer	Sequence (5′–3′)†	Amplified length
Full-length genome fragment 1	1F-Fwd	ACGCGAAAAAATGCATACCAAACCYA	1152 bp
	1F-Rev	GTGGATCTGGTAACACTGTAT	
Full-length genome fragment 2	2F-Fwd	TTGAGTGCACAGGATGCATTC	1041 bp
	2F-Rev	ACCTGTTCCATCTCAGCTT	
Full-length genome fragment 3	3F-Fwd	TTATGACCTGTTTGAAAGGAA	1007 bp
	3F-Rev	CCAATCCGACAAACTTCTC	
Full-length genome fragment 4	4F-Fwd	CCATGTTGCTCAGATGATAC	1228 bp
	4F-Rev	CTGCCTTTAGCAGTTTGAATGC	
Full-length genome fragment 5	5F-Fwd	TAAGCTCCGATGTGGACCATG	1152 bp
	5F-Rev	GGGTGGTCCCTTGCTCTGTGG	
Full-length genome fragment 6	6F-Fwd	GTGAACCAAATGTGTAAGCT	2156 bp
	6F-Rev	CACTACATGTGGACTCATAGAA	
Full-length genome fragment 7	7F-Fwd	TGCTCCAATTATTTCCAAGGAG	2103 bp
	7F-Rev	ACTTGTGGAAATCTTACAATCGTAGTT	
Full-length genome fragment 8	8F-Fwd	CTGTGACACTCTGAAAAGTCTAAC	1404 bp
	8F-Rev	GACAGATTGGATCAGACTGCAT	
Full-length genome fragment 9	9F-Fwd	GATAAGATACACAAGAAGAGACA	1814 bp
	9F-Rev	TGACCACATTATAAAATTCATTAGATGTTG	
Full-length genome fragment 10	10F-Fwd	GATTRAAAGGTCATCTGCTCA	1814 bp
	10F-Rev	CTGTTAAATGCARCCAAGAGAAT	
Full-length genome fragment 11	11F-Fwd	TAGGTGCTCATTAATTACTGATTTAA	1814 bp
	11F-Rev	TCTCAGCTTTATGGAATGGTAG	
Full-length genome fragment 12	12F-Fwd	GAATGATTCTGCTGAGTTTGTAAC	1814 bp
	12F-Rev	GTTTTAAAGCTWGAGAGGAAGAG	
Full-length genome fragment 13	13F-Fwd	TGTCTTGCTGGTCATTGGATC	1814 bp
	13F-Rev	GACTTAAGGCAGTGCTTACAG	
Full-length genome fragment 14	14F-Fwd	GAGTYTGGCATCAACTGATGCTA	1814 bp
	14F-Rev	ACGGGAAAAAAATACCCAAAAGAAGTC	
Glycoprotein fragment 1	G-1F-Fwd	GGAACCGAATGAGACCTGTAG	729 bp
	G-1F-Rev	AGAAGCCCTGATCACACCC	
Glycoprotein fragment 2	G-2F-Fwd	GCCCTGATCTTGTAACAATG	677 bp
	G-2F-Rev	GGTCAATTAAGGACTAGACCTCC	

Abbreviations: PPIV, porcine parainfluenza viruses; F, fragment; bp, base pair; G, glycoprotein

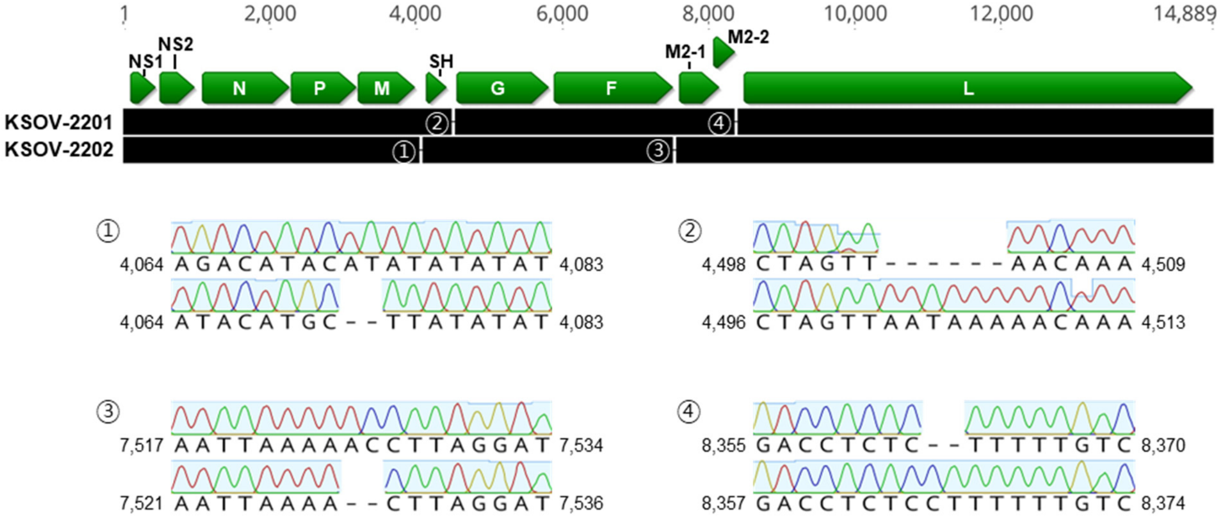

Figure S1. Alignment of complete genome sequences of KSOV-2201 and KSOV-2202. The top diagram represents the Cording regions of the SOV, including; non-structural protein 1 (NS1), NS2, nucleocapsid protein (N), phosphoprotein (P), membrane protein (M), small hydrophobic protein (SH), attachment glycoprotein (G), fusion protein (F), M2- 1, M2-2 and large protein (L). Among the aligned sequences, regions with insertions or deletions are represented by dashes, and each site is indicated by numbers. Chromatogram of nucleotides according to sequencing analysis was graphed at the insertion or deletion site of the corresponding number. The sequences were aligned in Geneious version 2022.0.2 via MAFFT.

```
SOV|Swine_pneumovirus_57   T.ATTCTCT.GGT.T.TGTTTG--.G.T.T.AA.-G...CAGCG.A.T.C.AT.AGTAGAGAG...TCGTGTG.TC..G   76
SOV|KSOV-2201*            TACGCGAAAAAAAACATAACACAACTATCA-CCCG-AAAAAGATAGGACAAGTAGGAATTGCTAGTCCTAAAGACCAGGA   78
SOV|KSOV-2202*            ..............................-....-.................GAAG..........C...........   78
MPV|mice_J3666             ...........TG.......A........A..T.-......T..........GAAG..........C.........A.   78
MPV|mice_15                ...........TG.......A........A..T.A......T..........GAAG..........C.........A.   79
CPnV|Bari/100-12          ............TG....C..A.C.C.C..CT...-......T..........GAAG..A.......TC..G......A.   79
CPnV|CP_13_TH/2015                                    ..CT...-......T..........GAAG..A.......TC..G......A.   51
CPnV|CP_82_TH/2016                                    ..CT...-......T..........GATG..A.......TC..G......A.   51
CPnV|SR1_TH/2016                                      ..CT...-......T..........GATG..A.......TC..G......A.   51
CPnV|SMU-2020-CB19                                                                         .TT......A.   11

SOV|Swine_pneumovirus_57  .-...T.TATG.G..AC.GGC.CTGCAGAT.T.G..............................T....   146
SOV|KSOV-2201*            TCATGGGCTGTAATGTGATTATGGAGCTTGACCATGGCGGTCGAACTGCATGGCTGGCTTTCCACACAACC   149
SOV|KSOV-2202*            .................T..C..A.......TT.....................................   149
MPV|mice_J3666            ...................G..........TT....T..A...G.............A.......T....   149
MPV|mice_15               ...................G..........TT....T..A...G.............A.......T....   150
CPnV|Bari/100-12          .......T...........G.........GTT....T..A...G........T....A.....T.T....   150
CPnV|CP_13_TH/2015        ...................G.........GTT....T..A...G........T....A.....T.T....   122
CPnV|CP_82_TH/2016        ...................G.........GTT....T..A...G........T....A.....T.T....   122
CPnV|SR1_TH/2016          .......T...........G.........GTT....T..AA..G........T....A.......T....   122
CPnV|SMU-2020-CB19        ......................................................................   82
```

Figure S2. Nucleotide alignment of 3' Leader and NS1 start position of MPV-like orthopneumoviruses. Based on the nucleotide sequence of KSOV-2201 strain, the dots (.) represent nucleotides that are identical. The horizontal lines (-) represent nucleotides changes due to insertion or deletion. Bases or dots in red indicate the start codon sequence (ATG) of NS1. The region marked with a yellow background means the part where Swine_pneumovirus_57 strain is different from other MPV-like orthopneumoviruses. The sequences were aligned in Geneious version 2022.0.2 via MAFFT. Stars (*) indicate the Korean SOV sequences described in this study.
